# Health promoting spirulina platensis nanoparticles induce early embryonic loss in heat-stressed rabbit does

**DOI:** 10.1038/s41598-026-66187-9

**Published:** 2026-08-26

**Authors:** Emtenan M. Hanafi, Ismail A. Elnady, Mohamed I. Shehab El-Din, Yasser H. A. Saber, Manal M. Ramadan, Reda M. S. Korany, Ghada H. Elsayed

**Affiliations:** 1 Department of Animal Reproduction and A.I, National Research Centre, Dokki, Giza, Egypt; 2 Department of animal production, Faculty of Agriculture, Al-AzharUniversity, Cairo, Egypt; 3 Department of flavor aromatic plants, National Research Centre, Giza, Egypt; 4 Department of Pathology, Faculty of Veterinary Medicine, Cairo University, Giza, Egypt; 5 Hormones Department, National Research Centre, Dokki, Giza, Egypt; 6 Stem Cell Lab, Centre of Excellence for Advanced Sciences, National Research Centre, Dokki, Giza, Egypt; 7 Department of Animal Reproduction and A.I, Veterinary Research Institute, National Research Centre, Dokki, Giza, Egypt

**Keywords:** Nano-*Spirulina platensis*, Heat stress, Cell programming, Embryo loss, Gene expression, Biochemistry, Biotechnology, Cell biology, Physiology

## Abstract

Heat stress poses a major constraint to rabbit production by impairing reproductive performance, compromising embryo survival, and increasing oxidative stress. Spirulina, known for its rich content of proteins, antioxidants, and bioactive compounds, has been proposed as a natural dietary supplement to mitigate these detrimental effects. The present study aimed to prepare nano size spirulina to evaluate its impact on reproductive performance and embryo survival of New Zealand White rabbit does under heat stress conditions. Sixty healthy pluriparous does were allocated into four groups: control (G1), heat stress (G2), and two heat-stressed groups (G3 and G4) supplemented with spirulina nanoparticles at two different levels. Reproductive traits, hormonal profiles, oxidative stress markers, proinflammatory cytokines and histopathological changes were assessed. The results revealed that heat stress significantly reduced fertility rates, progesterone levels, and increased embryonic loss, accompanied by oxidative damage and inflammatory responses. Supplementation with spirulina nanoparticles improved the general health condition of mothers and alleviated the adverse effects of heat stress; elevated antioxidant capacity, number of oocytes, conception and implantation. However, spirulina nanoparticle supplementation also exerted immunomodulatory effects that were associated with inadequate development of the placental spiral arteries and increased early embryonic loss in the treated groups. These findings suggest that spirulina nanoparticles may offer protective benefits against heat stress in rabbits, but caution was advised regarding using nanoparticles for does kept for breeding.

## Introduction

One of the most important environmental issues affecting rabbit production is heat stress. It decreases feed intake, weakens the immune system, inhibits the secretion of follicle-stimulating hormone (FSH) and luteinizing hormone (LH), and ultimately lowers reproductive performance^1,2^. Rabbits are especially vulnerable to high temperatures, which can cause delayed puberty, low conception rates, smaller litter sizes, and higher newborn mortality, particularly in the summer^3–5^. Heat stress’s detrimental effects are mostly caused by an overabundance of reactive oxygen species (ROS), which hamper sperm function, oocyte maturation, and fertilization, ultimately impeding embryo development^6,7^.

Nutritional therapies have been studied as possible methods to reduce heat stress and enhance rabbit reproduction. Due to its significant content of proteins, essential amino acids, fatty acids, vitamins, minerals, carotenoids, and phenolic compounds, the blue-green microalga *Spirulina platensis* (SP) has recently drawn interest as a dietary supplement^8^. It is regarded as a natural antioxidant that has been shown to improve ovarian function, puberty onset, and general reproductive health in addition to reducing oxidative stress^9^.

Rabbits’ fertilization and pregnancy stages, as well as the development of their embryos and placentas, are identical to those of humans^10^. At the molecular level, coordinated expression of regulatory genes that govern placental development, implantation, and embryo quality is necessary for the establishment and maintenance of a successful pregnancy. Pluripotency, lipid metabolism, and mitochondrial activity depend on genes like POU5F1, SOX2, NANOG, KLF4, GJA1, and PTGS2^11,12^. While, fetal allograft tolerance and maternal immune modulation depend on cytokine’s coordinated function^13^.

Nanotechnology have lately surfaced as a viable technique in animal nutrition at the molecular level. Compared to bigger particle types, nanoparticles have higher bioavailability, longer bloodstream persistence, and more focused effects^14^. Supplementing rabbits with nano-*spirulina platensis* (NSP) has been shown to enhance feed efficiency, vitality, ovarian function, and reproductive success^15^.

Based on this background, the present study aimed to evaluate the potential role of NSP in mitigating the adverse effects of heat stress on pregnant rabbit does with special reference to the expression of key genes regulating conception, placentation of fetal allograft, embryo development and immunomodulation during pregnancy course.

## Materials and methods

This study was reviewed and approved by the Institutional Ethical Committee for Medical Research of the National Research Centre, Egypt (Code No: 12–115; Ethical Approval No. 20170) for project 13,050,419. The ethical approval was in accordance with the provision of the relevant *Egyptian laws and Helsinki Declaration*, good medical and laboratory practice (GCP and GLP) arrive guidelines as well as the *institutional Animal Care and Use Committee* (IACUC) and medical plants guidelines and recommendation and *World Health Organization* (WHO) rules regarding the ethics of scientific research. The experiment was carried out in summer and was done for 20 days oral administration before fertilization, 30 days pregnancy course and one month after giving birth and till weaning.

### Preparation of the spirulina nanoparticles

**Fig. 1:**
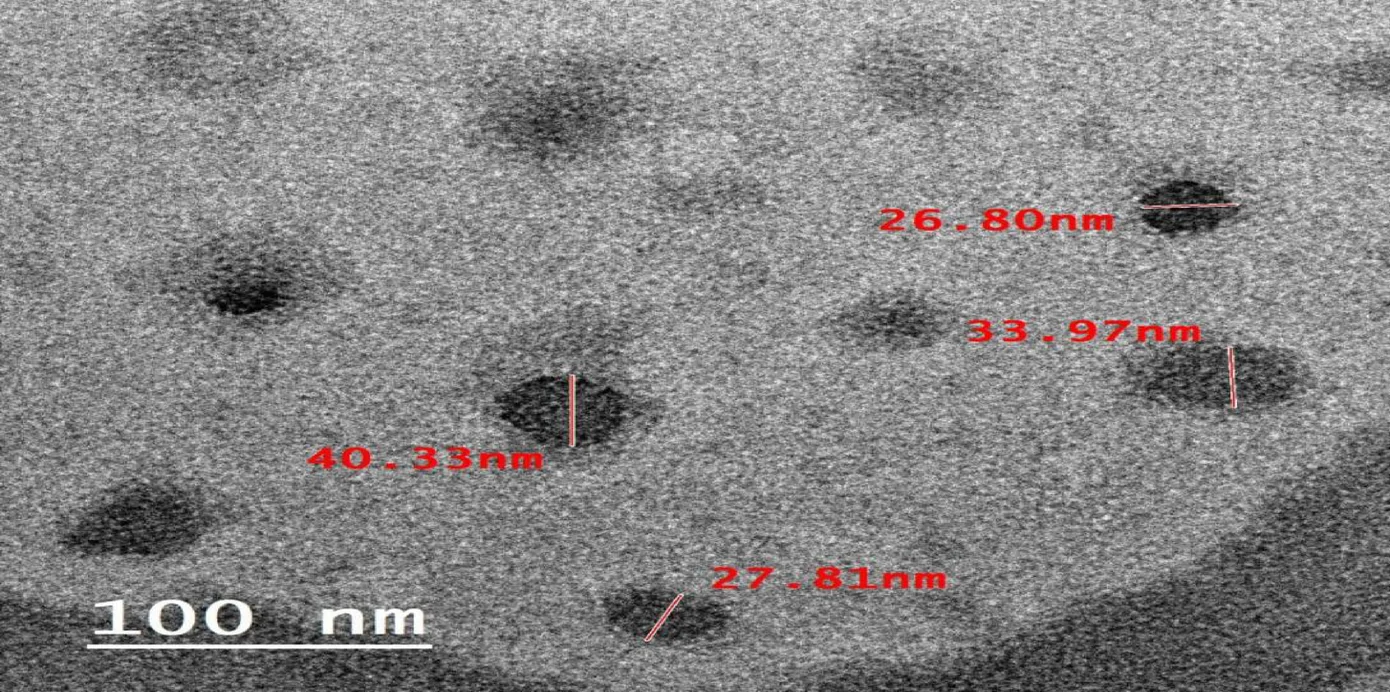
Photo of spirulinanano particles under transmission microscope.

Nanospirulina from NRC was reduced in size by sonication, using photon correlation spectroscopy with a Zeta Seizer Nano ZS (Malvern Instruments Inc., Southborough, MA) yielding particles of 26.8–42.33 nm (Fig. 1) with a ζ-potential of − 30.7, forming a stable, non-aggregating colloidal suspension^16^.

### Cytotoxic effect of nanospirulina on human fibroblast cell line

The biosafety of the prepared nano spirulina was evaluated on the human fibroblast BJ1 cell line. (CRL-2522) was purchased from ATCC (American Type Culture Collection; LGC Promochem AB, Boras, Sweden). Results showed high biocompatibility, with only 13.5% cell death at 100 ppm, reflecting a normal physiological response^17^.

## Biological experiment

### Experimental design

Sixty healthy pluriparous New Zealand White does were purchased from rabbit farm of AL- Azhar University, Cairo, Egypt. Animals were equally divided into four groups (G1–G4) and housed individually. G1 was maintained under ambient conditions, while G2, G3 and G4 were exposed to heat stress. G1 and G2 received a basal diet, whereas G3 and G4 received the same diet and orally supplemented with spirulina nanoparticles suspension (15 and 35 mg/kg B.W., respectively). Animals received the treatment for 50 consecutive days, including 20 days before artificial insemination and throughout the 30-day gestation period. Reproductive outcomes were recorded until weaning. Health status, feed intake, feed efficiency ratio, reproductive parameters (CLs, conception rate, number placentation sites, gestation length and litter outcomes) were recorded.

### Environmental conditions in the controlled chamber

The experiment was conducted in environmentally controlled chambers under heat stress conditions. Rabbits in the heat-stressed groups (G2, G3, and G4) were continuously housed under these environmental conditions throughout the experimental period (24 h/day). Air temperature and relative humidity were continuously monitored, and the temperature–humidity index (THI) was calculated as THI = Tdb – (0.31–0.31 RH) (Tdb – 14.4), where Tdb is the dry-bulb temperature (°C) and RH is the relative humidity. Heat-stressed groups (G2, G3, and G4) experienced higher ambient temperatures and THI values (33.99–36.78), exceeding the critical thermal comfort limit for rabbits, while the control group (G1) remained within the thermoneutral range (24.81–27.40), ensuring normal physiological and reproductive responses (Table 1).

**Table 1:** Ambient temperature (C°), relative humidity (RH%) and THI values in different weeks of the experimental period under heat stress.

Weeks	C°		RH%		THI
	Minimum	Maximum	Minimum	Maximum	Mean
The condition of heat and humidity of G2,G3 and G4					
W1	26 ± 0.42	37 ± 0.5	38 ± 2.20	65 ± 1.4	34.54 ± 0.55
W2	28 ± 0.96	39 ± 1.79	49 ± 3.83	71 ± 1.97	36.78 ± 1.54
W3	26 ± 0.26	36 ± 0.89	57 ± 4.63	70 ± 3.51	33.99 ± 0.72
W4	25 ± 0.34	37 ± 0.26	34 ± 0.28	73 ± 2.25	35.10 ± 0.16
Normal conditions for control (G1)					
W1	23 ± 0.44	32 ± 0.52	44 ± 2.20	55 ± 1.42	25.68 ± 0.54
W2	24 ± 0.85	32 ± 1.75	45 ± 3.81	64 ± 1.95	27.40 ± 1.57
W3	25 ± 0.24	32 ± 0.86	56 ± 4.62	60 ± 3.54	26.25 ± 0.70
W4	26 ± 0.32	31 ± 0.27	35 ± 0.27	53 ± 2.26	24.81 ± 0.14

### Insemination of females

Semen was collected from six sexually mature New Zealand White bucks using an artificial vagina for the artificial insemination of the female rabbits. Immediately after collection, the gel fraction was removed, and the semen was incubated at 37 °C until individual evaluation. Tris-citrate extender (tris 3.028 g/100 mL, citric acid 1.69 g/100 mL, glucose 0.931 g/100 mL, 20% egg yolk, 25 mg of gentamicin, and 50000 IU of penicillin per 100 mL) was used to dilute pooled semen. At least 35 × 10^6^ spermatozoa, > 70% motility and > 75% live sperm were present in each insemination dosage (1 mL prolonged semen). Before insemination, 0.25 mL of Receptal (1 µg of buserelin acetate; Hoechst Marion Roussel, Madrid, Spain) was injected intramuscularly into each female to stimulate ovulation.

### Pregnancy diagnosis and reproductive evaluation

Pregnancy was diagnosed using abdominal palpation and transabdominal ultrasonography^18^. Abdominal palpation was performed to confirm conception, while ultrasonographic examination was used to confirm pregnancy and monitor its viability throughout gestation. At 10th day of pregnancy, eight does from each group (G1–G4) were euthanized under ketamine hydrochloride anesthesia (45 mg/kg BW, IM) for clinical and laboratory investigations. The remaining seven does in each group were allowed to complete gestation and were used for evaluating litter performance at birth and weaning. The numbers of corpora lutea (CL) and placentation sites were recorded, whereas the remaining animals were allowed to complete gestation. Litter size, the number of live and dead kits, and litter weights at birth and weaning were subsequently recorded.

### Blood biochemistry

Ten days after insemination, Animals were euthanized under anaesthesia with ketamine hydrochloride (45 mg/kg, IM). blood samples were taken from each group (*N* = 8), centrifuged at 600 ×g for 20 min, and the separated serum was stored at -20 °C to measure the levels of hormones (FSH, LH, progesterone), cytokines (TGF-β2, INF-γ, TNF-α, IL-1β), and oxidative markers (MDA, SOD, and Catalase) using an ELISA commercial kit (SUNLONG, China). The assay’s sensitivity was 0.05 ng/mL for rabbit P4, 0.1 ng for FSH, 0.01 ng/mL for LH, 0.5 pg/mL for IN-Fγ, 0.1 pg/mL for TNF-α, 0.6 pg/ml for TGF-β2, and 0.7 pg/mL for IL-1β. For every ELISA kit, the intra-assay and inter-assay precision was less than 12%. Additionally, colorimetric techniques (Bio diagnostic commercial kits) were used to determine the levels of cholesterol, ALT, AST, creatinine, total protein, albumin, and globulin in serum samples.

### Quantitative real-time PCR (qRT-PCR) analysis of gene expression

Using the RNeasy mini-Kit (Cat. No. 74104, Qiagen, Germany), RNA was extracted from rabbit uterus tissues. The concentration and purity of the collected RNA were then measured using a Nano Drop 2000 spectrophotometer (Thermo Fisher Scientific, USA). Following the manufacturer’s instructions, the Revert Aid First Strand cDNA Synthesis Kit (Cat. No. K1621, Thermo Fisher Scientific, USA) was used to transform the RNA from each treatment into first-strand cDNA. Table 2 lists specific primer sequences (Invitrogen, USA). POU5F1, SOX2, NANOG, KLF4, GJA1, PTGS2, TNF-α, IFN-γ, and TGF-β gene expression levels were estimated using the 2^−ΔΔCT^ technique^19^ and standardized with respect to GAPDH transcript using Maxima SYBR Green qPCR Master Mix (2X) (Cat. No. K0221, Thermo Fisher Scientific, USA). For a total of 40 cycles of amplification, the reaction conditions were 95 °C for 10 min, 95 °C for 15 s, 55° C for 30 s, and 72 °C for 30 s. Gene expression was quantified using the DNA Technology Detecting Thermo cycler DT Lite 4S1 (Russia).

**Table 2:** QRT-PCR primers for the studied genes.

Gene	Forward primer (5′-3′)	Reverse primer (5′-3′)	GenBank Accession No
GAPDH	CGCTGGGACGGGGTGCCCTTCATC	CGCCAGGCCTCCCGCAGTTCATCA	XM_583628.4
POU5F1	AAGCGGGGACCCTCGTGCAG	TCTGGCGCCGGTTACA	KJ708484
SOX2	AGCCCCAAGATGCACAACTC	CTCCGGGAAGCGTGTACTTA	KJ939365
NANOG	CCTCTCCGCTTCCTTCCT	CTGTTTGTAGCTAAGGTTCAGGAGG	KJ708482
KLF4	AGCGGGAAGGGAGAAGACAC	AGGACGAGGAAGAGGCAGAC	KM267140
GJA1	TGCCTTTCGTTGTAACACTCA	AGAACACATGAGCCAAGTACA	NM_001198948.1
PTGS2	TCCAAGCTGGCCTCACTGATGG	AGCATGTGTGTGGCCCGACTTG	NM_001082388.1
TNF-α	GTCTTCCTCTCTCACGCACC	TGGGCTAGAGGCTTGTCACT	M12845
IFN-γ	TTCTTCAGCCTCACTCTCTCC	TGTTGTCACTCTCCTCTTTCC	D84216
TGF-β	CAGTGGAAAGACCCCACATCTC	GACGCAGGCAGCAATTATCC	NM_001082660

### Histopathological assessment

Ovarian and uterine samples were gathered, fixed in 10% neutral buffered formalin, cleaned, dehydrated, and embedded in paraffin. For histological analysis, the paraffin-embedded blocks were sectioned at a thickness of 5 microns and stained with hematoxylin and eosin^20^.

### Statistical analysis

The number of samples was 8 for each group. Statistical results were displayed as mean ± SEM. One-way analysis of variances (one-way ANOVA) and the Tukey Kramer multiple comparison test were used to determine whether there was a statistically significant difference between the groups. Graph Pad Prism version 8 was utilized for data analysis. For every test, a significant difference was defined as *p* < 0.05.

## Results

### Pregnancy assessment

Pregnancy examination revealed normal embryonic development in the control groups (G1 and G2), whereas embryonic resorption was detected in the heat-stressed groups (G3 and G4) after gestational day 10. The reproductive performance, including the number of corpora lutea, placentation sites, gestation length, litter size, and litter weight, is summarized in Table 3.

**Table 3:** Effect of heat stress and nanospirulina on pregnant mothers and newborns.

Parameters	G1	G2	G3	G4
Conception rate %	0.93	0.86	0.80	0.95
Number of CL (n)	8.2 ± 0.23	6.4 ± 0.12^a^	8.2 ± 0.11^b^	10.6 ± 0.15^ab^
Number of placentation sites	8.2 ± 0.11	5.4 ± 0.21^a^	8.0 ± 0.13^b^	9.4 ± 0.15^ab^
Gestation length	30.5 ± 0.20	31.54 ± 0.2	< 10 days	< 10 days
Born alive (n)	6.8 ± 0.13	5.4 ± 0.21^a^	………	……………
Born dead (n)	0.58 ± 0.2	0.70 ± 0.1^a^	-----------	------------
Litter size at birth ( cm)	7.14 ± 0.20	6.31 ± 0.13^a^	------------	------------
Litter weight at weaning (gm)	304 ± 11.1	300 ± 16.0^a^	……….	……….

Results were mentioned as mean ± SEM (N 15). Different superscript letters (a, b) indicate significant differences among the four experimental groups (*p* < 0.05).

### Effect of heat stress on the does’ health condition

**Fig. 2:**
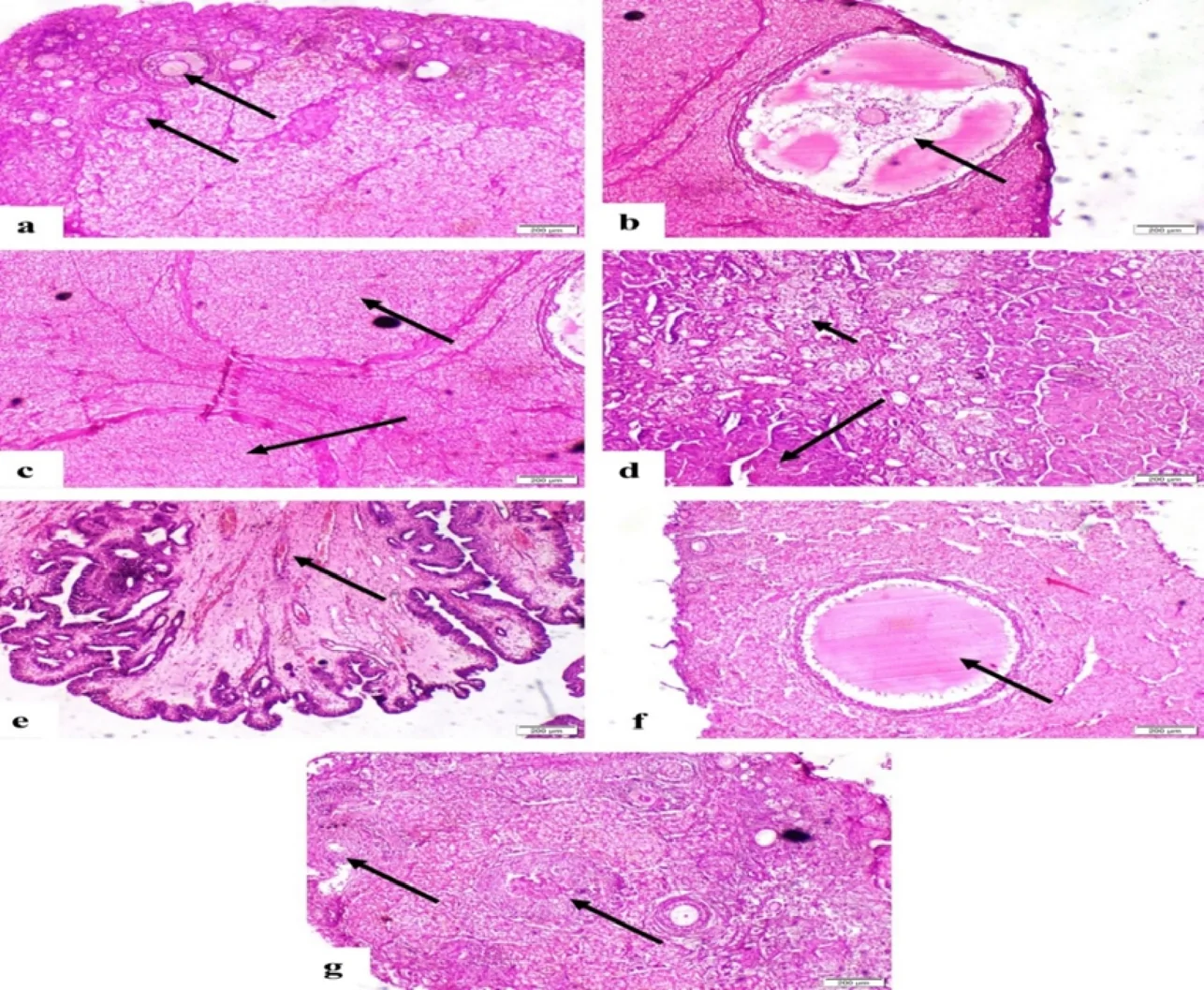
Photomicrograph, ovaries, uterus and placenta of control group (scale bar 200 μm) (**a**) Control group ovary (G1) showing numerous ovarian follicles (arrows). (**b**) Control group ovary (G1) showing mature ovarian follicle (arrow). (**c**) Control group (G1) ovary showing corpora lutea (arrows). (**d**) Control group (G2) uterus showing mature placenta composed of labyrinth (long arrow), basal zone (short arrow). (**e**) Control group (G1) uterus showing mature and highly vascular placental villi (arrow). (**f**) G2 ovary showing ovarian follicular cysts (**g**) G2 ovary showing atretic and necrosed follicles (arrows).

Results revealed (Table 4) that animals kept under heat stress showed low feed intake and feed efficiency ratio (FER) compare to normal control group. Also, heat stress (Table 3) reduced the number of CLs, conception, number of litter born live, size and weight at weaning in G2 compared to G1. These results were confirmed by histopathology finding where the ovaries of G2 showed cystic and atretic follicles (Fig. 2f, g) compared to normal G1 (Fig. 2b and c).

**Table 4:** Feed intake (FI) and feed efficiency ratio (FER) of animals under heat stress (HS).

groups	FI at 1st week	FI at 2nd week	FI at 3rd week	FI at 4th week	FER
G1 (C normal)	1277.4 ± 20.79	1498.0 ± 17.4^a^	1610.0 ± 21.53^a^	1716.4 ± 19.62^a^	0.100
G2 (C + HS)	1252.4 ± 31.88	1260.0 ± 41.54^b^	1296.4 ± 37.02^b^	1422.4 ± 42.0^ab^	0.072
G3(nsp 15 mg + HS)	1252.4 ± 25.77	1200.0 ± 21.34^b^	1256.4 ± 17.0^b^	1322.4 ± 22^b^	0.107
G4(nsp 30 mg + HS)	1191.4 ± 19.73	1202.4 ± 42.06^b^	1262.4 ± 22^ab^	1312.4 ± 12^b^	0.120
*p* < 0.05	0.13	0.001	0.001	0.001	

Results were mentioned as mean ± SEM. Different superscript letters (**a**,** b)** indicate significant differences among the four experimental groups (N 15) (*p* < 0.05).

**Fig. 3:**
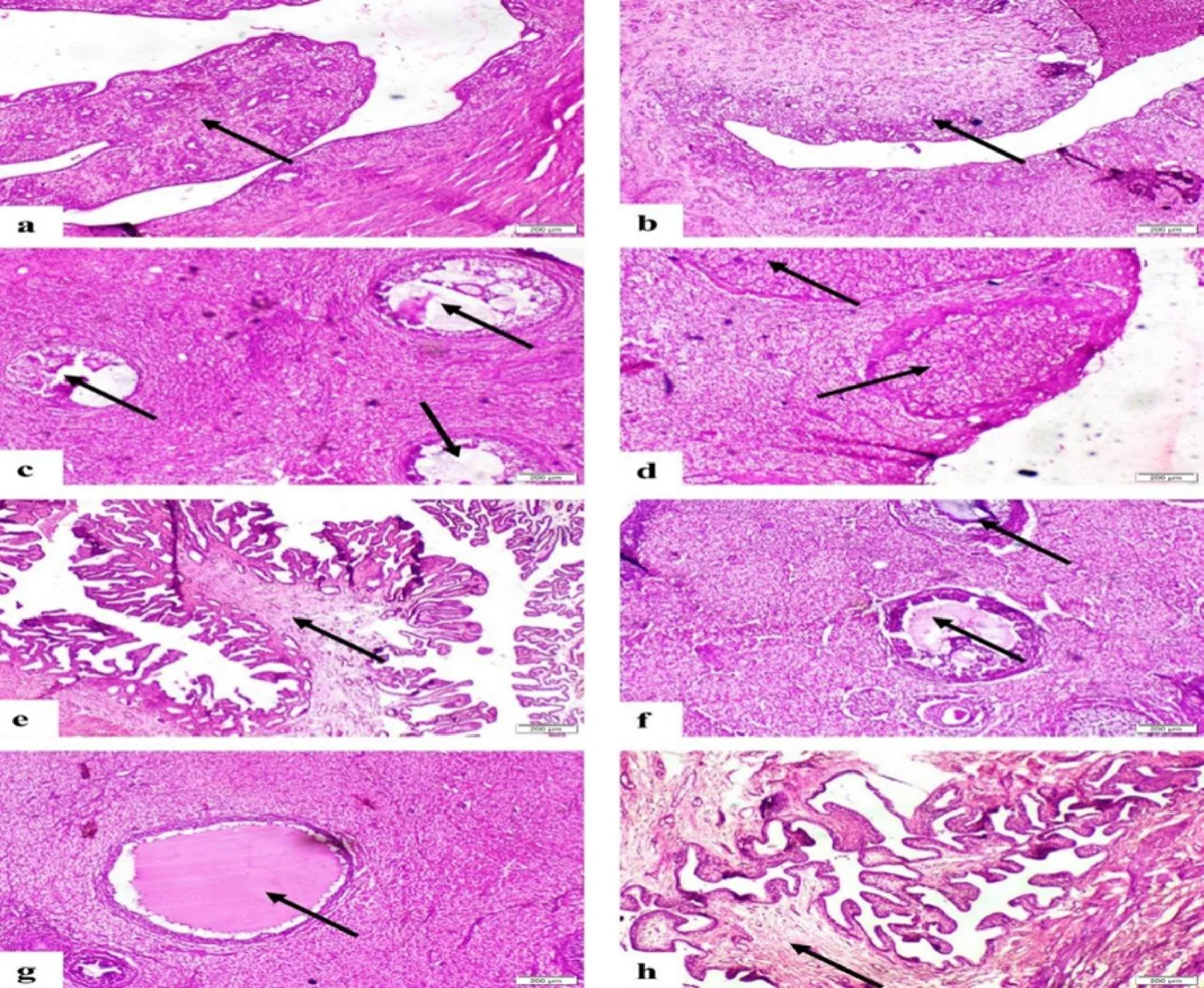
Photomicrograph, ovaries and uterus of rabbit does Kept under heat stress (scale bar 200 μm) (**a**) G2 uterus showing hyperplasia of endometrium with papillary in folding into the uterine lumen (arrow). (**b**) G2 uterus showing few and small endometrial glands (arrow). (**c**) G3 ovary showing atretic follicles (long arrows) with few healthy one (short arrow). (**d**) G3 ovary showing few small corpora lutea (arrows). (**e**) G3 uterus showing immature avascular placental villi (arrow). (f) G4 ovary showing mature follicle (short arrow) and destruction and necrosis of others (long arrow). (**g**) G4 ovary showing cystic ovarian follicles (arrow). (**h**) G4 uterus showing immature avascular placental villi (arrow).

Also, the examination of placenta of rabbit does kept under heat stress (G2) showed hyperplasia of endometrium with papillary infolding into the uterine lumen and uterus showed few and small endometrial glands (Fig. 3a, b) compared to control group kept under ambient temperature (G1) where the uterus showed mature placenta composed of labyrinth basal zoon and mature highly vascular placental villi (Fig. 2d, e).

**Fig. 4:**
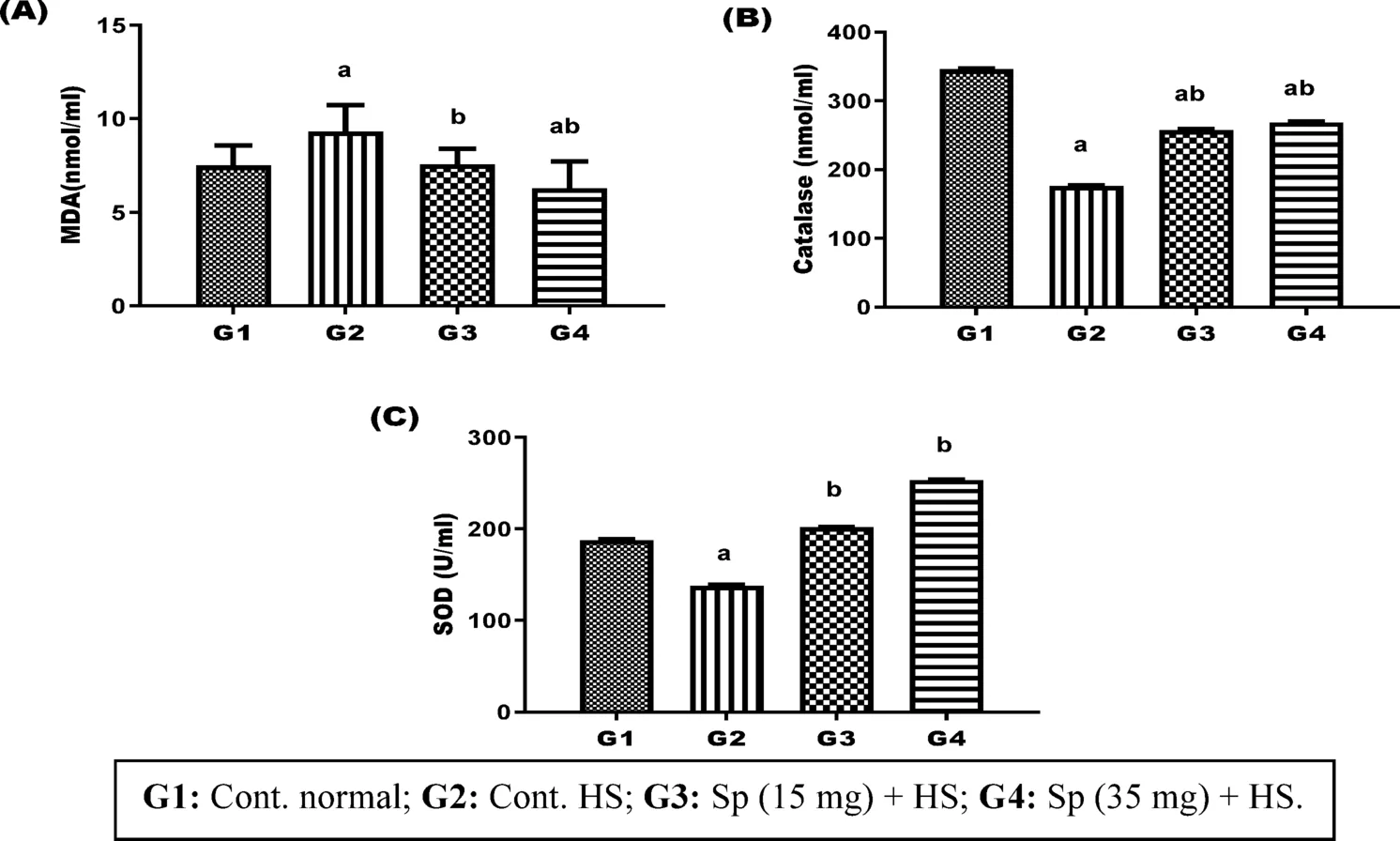
(**A**–**C**). Oxidative markers of rabbit does kept under heat stress and given nanospirulina. Data are represented as mean ± SEM (*n* = 6). Different superscript (**a**, **b**) indicate significant differences among the four experimental groups (*p* < 0.05).

**Fig. 5:**
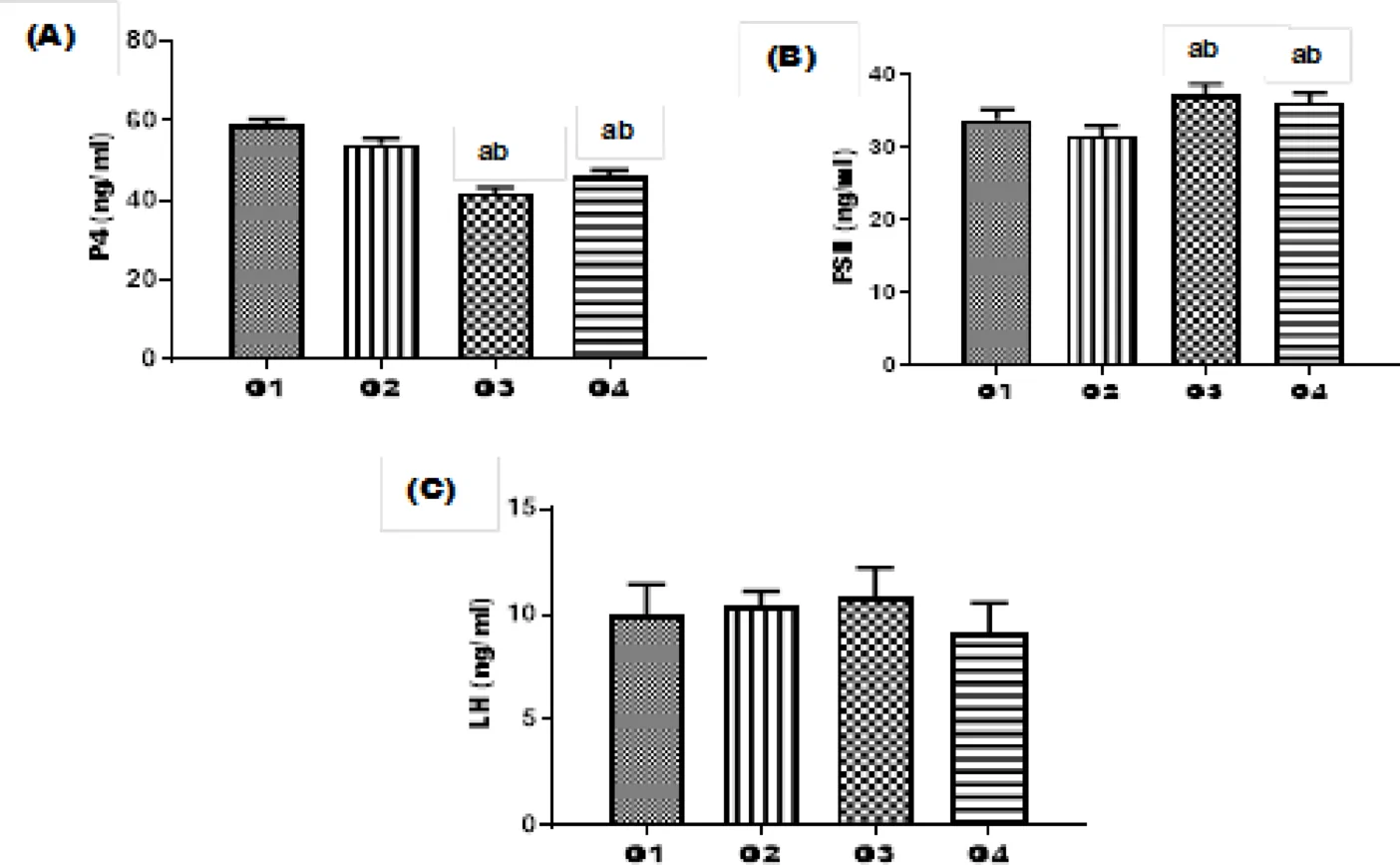
(**A**–**C**). Serum reproductive hormones of rabbit does kept under heat stress and given nanospirulina. Data are represented as mean ± SEM (*n* = 6). Different superscript (**a**, **b**) indicate significant differences among the four experimental groups (*p* < 0.05).

**Fig. 6:**
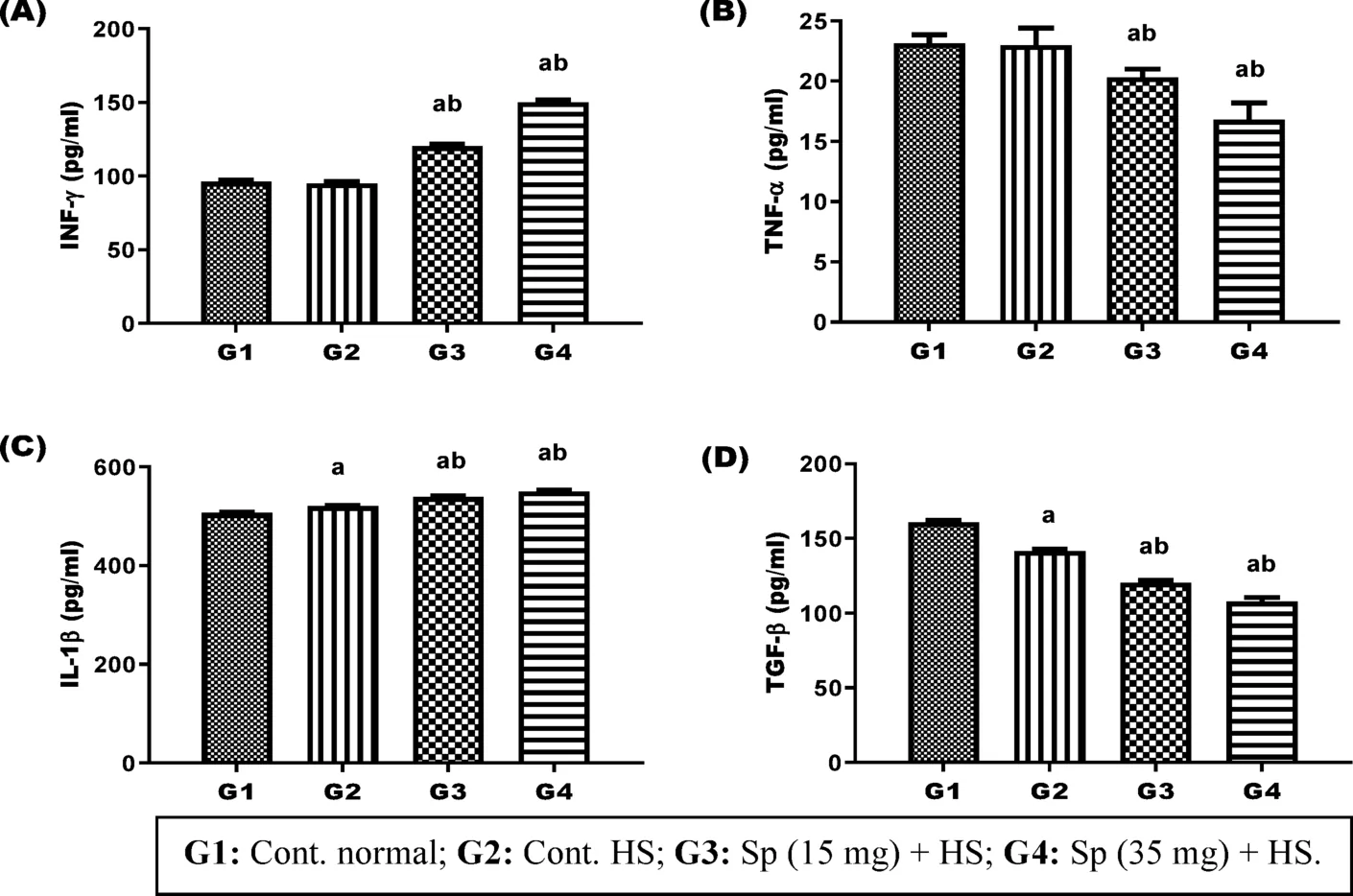
(**A**-**D**). Serum inflammatory cytokines in rabbit does kept under heat stress and given nanospirulina. Data are represented as mean ± SEM (*n* = 8). Different superscript (a, b) indicate significant differences among the four experimental groups (*p* < 0.05).

The results showed that does in the heat stress group (G2) exhibited moderate health status and vitality, accompanied by significantly elevated serum MDA levels and reduced SOD and catalase activities (Fig. 4A–C). In addition, slightly reduced levels of progesterone, follicle-stimulating hormone (FSH), and luteinizing hormone (LH) were observed in G2 compared with the control group (G1) (Fig. 5A–C). These hormonal changes were associated with high level of serum inflammatory cytokines INF-γ, IL-1β, and TNF-α (Fig. 6A-D).

**Fig. 7:**
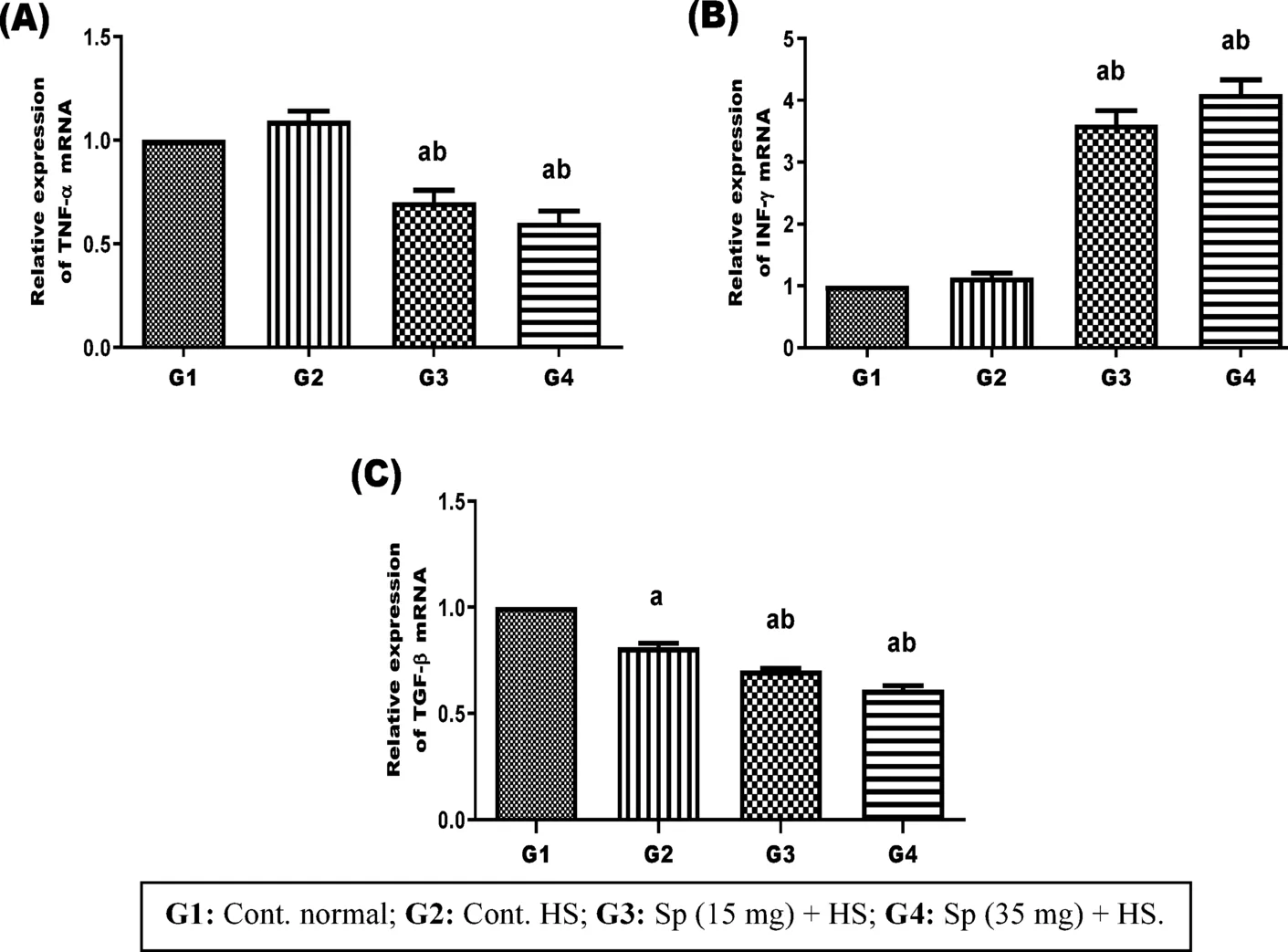
(**A**-**C**). Quantitative real-time PCR analysis of TNF-α, IFN-ɣ, and TGF-β gene expression in the studied groups. Data are represented as mean ± SEM (*n* = 8). Different superscript letters (**a**, **b**) indicate significant differences among the four experimental groups (*p* < 0.05).

**Fig. 8:**
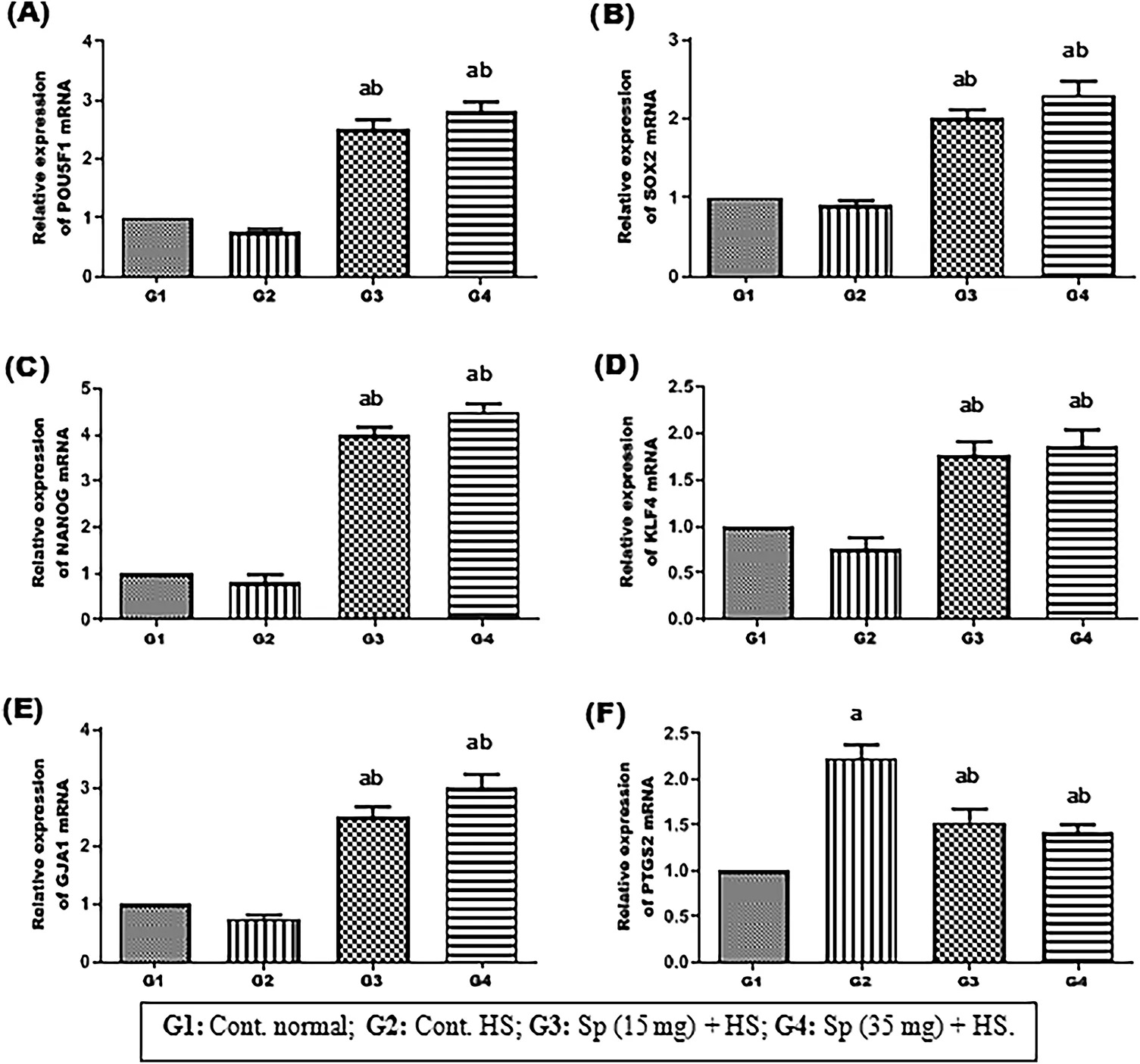
(**A**-**F**). Quantitative real-time PCR analysis of POU5F1, SOX2, NANOG, KLF4, GJA1, and PTGS2 gene expression in the studied groups. Data are represented as mean ± SEM (*n* = 8). Different superscript letters (**a**, **b**) indicate significant differences among the four experimental groups (*p* < 0.05).

Consistent with these findings, the mRNA expression levels of the inflammatory cytokines TNF-α and IFN-γ were significantly upregulated in G2 by approximately 9% and 14%, respectively, while TGF-β was significantly decreased by about 19% compared with G1 (Fig. 7A–C). Analysis of uterine samples revealed that the expression of pluripotency-related genes (POU5F1, SOX2, NANOG, KLF4, and GJA1) was slightly increased in G2 by approximately 25%, 10%, 20%, 25%, and 25%, respectively; however, these levels remained lower than those observed in G1 (Fig. 8A–E). Furthermore, G2 group exhibited a significant upregulation (*p* < 0.05) of PTGS2 expression, reaching approximately 123% higher than that of the G1 group (Fig. 8F).

### Effect of nano-spirulina on rabbits does kept under heat stress

The results presented in Table 4 showed improved feed intake and FER in groups kept under HS and given nanospirulina (G3, G4) compared to G2 control group. Also, the number of CLs and placentation sites (Table 3) were significantly higher in groups G3 and G4, which received nano-spirulina at doses of 15 and 35 mg/kg body weight, respectively, compared with both G1 (control normal) and G2 (control under heat stress). In addition, analysis of reproductive hormones revealed elevated levels of FSH in treated groups compared to control (Fig. 5B).

The present study also demonstrated the antioxidant and anti-inflammatory effects of nanospirulina (Figs. 4 and 6), as evidenced by a significant increase in serum antioxidant enzyme (catalase and SOD) and reduction in serum TNF-α levels (Fig. 6B). Consistently, TNF-α gene expression was significantly down regulated in G3 and G4 by approximately 36% and 45%, respectively, compared with G2 (Fig. 7A). Moreover, biochemical analysis confirmed the biosafety of nanospirulina on liver and kidney functions and showed a reduction in serum cholesterol levels (Table 5).

**Table 5:** Serum biochemical parameters of pregnant does under heat stress and nano-spirulina supplementation.

	G1	G2	G3	G4
Cholesterol mg/dl	44.79 ± 15.81	33.42 ± 2.06^a^	39.32 ± 2.15^a^	38.51 ± 2.15^a^
ALT U/L	30.827 ± 0.31	36.817 ± 2.27^a^	37.6 ± 4.47^a^	38.620 ± 5.17^ab^
AST U/L	14.56 ± 1.04	24.9 ± 8.56^a^	20.31 ± 4.54^ab^	20.13 ± 6.07^ab^
Creatinine mg/dl	2.60 ± 0.66	5.20 ± 0.489^a^	3.50 ± 1.88^ab^	3.40 ± 0.90^ab^
Total protein g/dl	3.13 ± 0.20	3.19 ± 0.38	3.33 ± 0.3	3.62 ± 0.17
Albumin g/dl	2.51 ± 0.09	2.99 ± 0.39	2.13 ± 0.65^b^	1.95 ± 0.38^b^
Globulin g/dl	0.62 ± 0.23	0.57 ± 2.06^a^	1.26 ± 1.12^ab^	1.66 ± 0.49^ab^

G1 control normal group, G2 control under heat stress, G3 administered nanospirulina 15 mg/kg under heat stress, G4 given nanospirulina 35 mg/kg under heat stress. Results were mentioned as mean ± SEM (N 8). Different superscript letters (a, b) indicate significant differences among the four experimental groups (*p* < 0.05).

Quantitative gene expression analysis (Fig. 8A–E) revealed significant upregulation of pluripotency-associated genes involved in embryonic cell division and development from fertilization to implantation in the spirulina-treated groups. Specifically, expression levels of POU5F1 (~ 233% and 273%), SOX2 (~ 122% and 156%), NANOG (~ 400% and 463%), KLF4 (~ 135% and 148%), and GJA1 (~ 233% and 300%) were significantly increased in G3 and G4, respectively, relative to the G2 group In contrast, the treated groups G3, G4 showed significant downregulation in PTGS2 (~ 31% and 36%) in a dose-dependent manner (Fig. 8F).

The histopathological findings showed the evidence of immature avascular placental villi in G3 and G4 administered nanospirulina (Fig. 3e and h). Also, ovarian tissue of these groups showed some mature, cystic and necrosed follicles (Fig. 3f and g).

### Effect of heat stress on embryo of control mothers

**Fig. 9:**
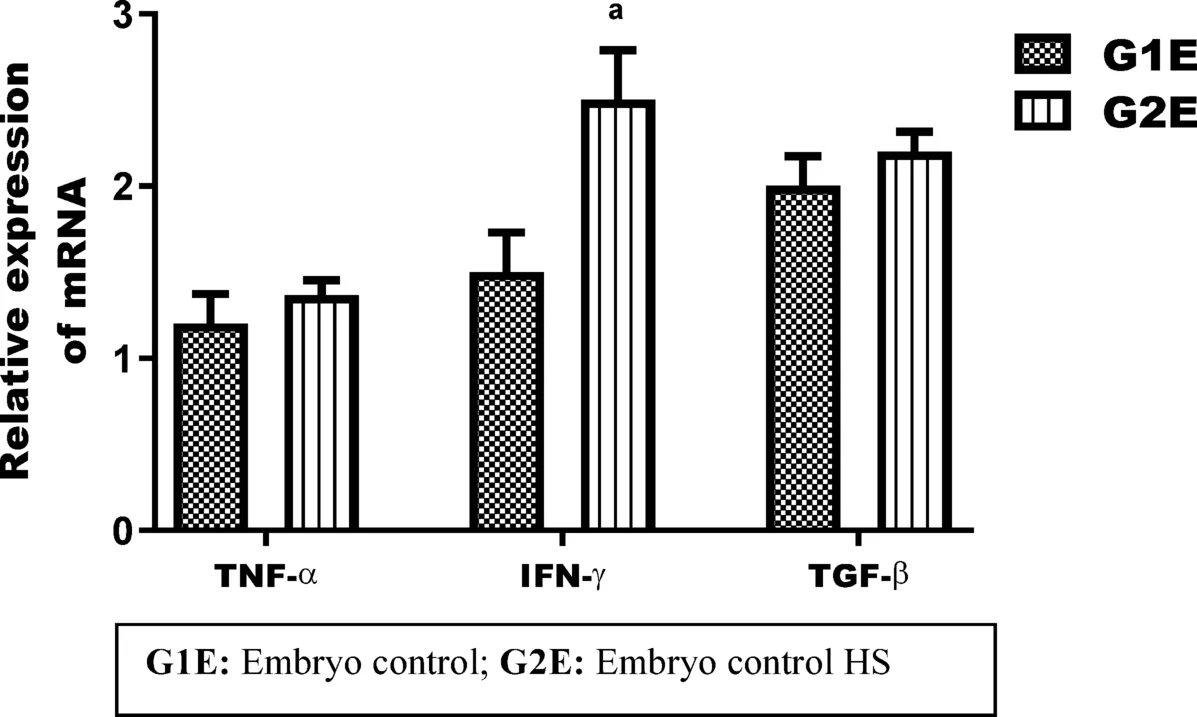
Quantitative real-time PCR analysis of TNF-α, IFN-ɣ, and TGF-β gene expression in the embryo groups. Data are represented as mean ± SEM (*n* = 8). Superscript ***a*** indicates a significant difference compared with the embryo control group (G1E) (*p* < 0.05).

**Fig. 10:**
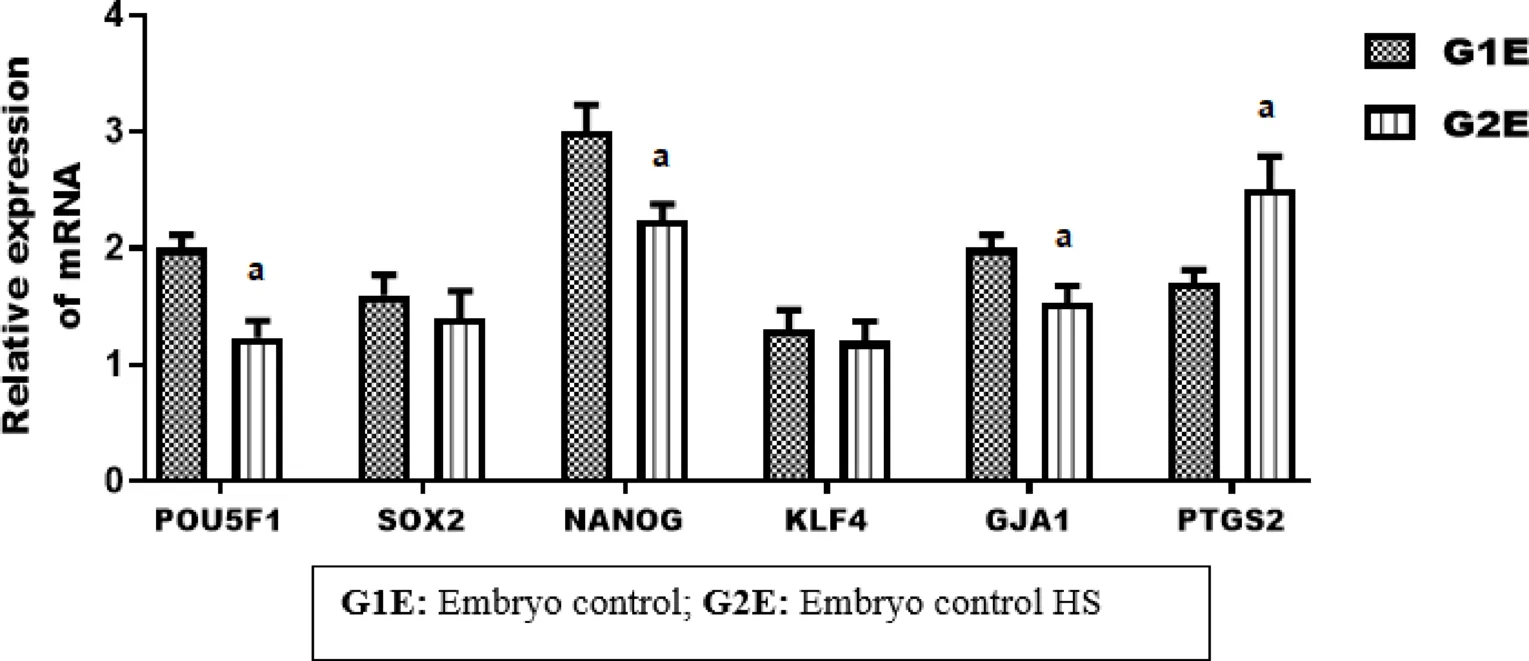
Quantitative real-time PCR analysis of POU5F1, SOX2, NANOG, KLF4, GJA1, and PTGS2 gene expression in embryo groups. Data are represented as mean ±SEM (*n* = 8). Superscript ***a*** indicates a significant difference compared with the embryo control group (G1E) (*p* < 0.05).

In spite of heat stress female rabbits of G2 completed the pregnancy course but the size, weight and viability of the newborns were lower compared to G1 (Table 3). The gene expression levels of INF-γ showed significant higher levels in the G2 of rabbit embryos (G2E) relative to G1 embryo (G1E) (Fig. 9). Also, PTGS2 gene expression was upregulated in G2E compared to G1E (Fig. 10). Furthermore, the expression of the pluripotency genes POU5F1, SOX2, NANOG, KLF4, and GJA1 (Fig. 10) in embryo samples was less than that in the normal control group (G1E).

### Effect of nano-spirulina on embryos viability

The current study showed early embryonic loss in groups of animals kept under heat stress and administered nano-spirulina (G3 and G4), despite the presence of placentation site at higher number than control groups. The histopathological finding of placenta of such groups showed immature avascular placental villi (Fig. 3e and h) and this may be the cause standing behind fetal death and desorption.

Gene expression analysis (Fig. 7B) demonstrated upregulation of IFN-γ gene expression by approximately 215% and 260% in the nano-spirulina–treated groups (G3 and G4), respectively. Consistently, elevated serum levels of the Th1 cytokines (IFN-γ and IL-1β) were observed in these groups (Fig. 6A, C). In contrast, nano-spirulina treatment significantly reduced serum TGF-β levels (Fig. 6D) and downregulated TGF-β gene expression by approximately 14% and 26% in G3 and G4, respectively, compared with the normal control group (G1) and the heat stress group (G2) (Fig. 7C).

## Discussion

Heat stress (HS) adversely affects reproductive performance in rabbits by delaying puberty, reducing conception rates and litter size, and increasing neonatal mortality, particularly during the summer season^4,5^. Exposure to heat stress enhances the production of free radicals^1^, which negatively influences the maturation and fertilization of oocytes^6^. Nano sized particles were reported to be more targeting at lower dose than ordinary sized particles. The prepared nano sized *Spirulina platensis* was reported as rich source of antioxidants and was presumed to alleviate the negative effect of heat stress on pregnant rabbit does.

The present study revealed that rabbits exposed to heat stress exhibited reduced feed intake and feed efficiency ratio (FER). However, nanospirulina supplementation improved FER, likely due to its antimicrobial activity, which modulates intestinal microbiota and conserves metabolic energy. In addition, nanospirulina increases the surface area of intestinal villi, thereby enhancing nutrient absorption^15^. Moreover, heat stress significantly elevated MDA levels and inflammatory cytokines, leading to oxidative stress that disrupts cellular metabolic processes. These alterations adversely affected the immunological state and reproductive efficiency of the animals^3^, as reflected in the present study by reduced numbers of CLs, conception, newborns lives, litter size and weight, increased cystic, atretic, and necrotic ovarian follicles.

Spirulina nanoparticles supplementation improved the general health status of rabbit does and demonstrated biosafety with respect to organ function. It also enhanced ovarian function, as evidenced by increased folliculogenesis and elevated reproductive hormone levels. Oocytes numbers, conception rate, number of placentation sites. Furthermore, nano spirulina significantly increased antioxidant capacity and suppressed inflammatory markers. These effects are consistent with previous reports describing spirulina as a potent health enhancer rich in vitamins, minerals, antioxidants, flavonoids, and polyphenols that alleviate heat stress-induced damage^21^.

Heat stress was reported to have hazardous effect on embryos at the 2 and 4 cell stages, particularly during days 3–5 of pregnancy in rabbits^22^. In contrast, embryos at later stages (8–16 cells to blastocyst) demonstrate greater resistance to thermal stress, and the expression of pluripotency-related genes (POU5F1/OCT4, SOX2, and KLF4) and implantation-related genes (NANOG and GJA1) remains largely unaffected^23^. These observations are consistent with the present findings, which showed a non-significant reduction in pluripotency gene expression between G2 and G1 groups.

Nano spirulina treatment upregulated the expression of pluripotency associated genes. Similar findings have been reported in cloned porcine blastocysts, where spirulina enhanced the expression of pluripotency-related genes such as POU5F1, DPPA2, and NDP52^24^.

Prostaglandins (PTGs) play an essential role during early pregnancy, including ovulation, fertilization, implantation, and decidualization. In the current study, PTGS2 gene expression was significantly elevated in G2 compared with G1 group. Dysregulation of PTGS2 expression is linked to ovarian cysts, fewer healthy follicles, and reduced corpora lutea^25^. This upregulation coincided with increased ROS production and inflammatory cytokine levels, as well as histopathological findings of follicular cysts, atresia, and degeneration, with fewer corpora lutea compared with G1 group. Despite the beneficial effects of nano spirulina, early embryonic loss was observed in heat-stressed groups receiving nano-spirulina (G3 and G4) with presence of placental remnants.

The histopathological examination revealed ill developed avascular placental villi. This outcome may be related to immunomodulatory effect of nanospirulina that can skew the Th2 polarization to Th1 like state by enhancing NK activity evidenced by increased gene expression of INF-γ and IL-1β (Th1 cytokine) and decreased TGF-β levels (Fig. 5). These findings are consistent with previous reports indicating that Spirulina polysaccharide adjuvant therapy enhances mononuclear cell activity, leading to increased production of pro-inflammatory cytokines in mice^26^. Moreover, Spirulina has been shown to stimulate macrophages to secrete IL-12, which subsequently promotes IFN-γ production by natural killer (NK) cells and T lymphocytes^27^ and subsequently regulating follicular development, ovulation, and apoptosis, as well as mediating inflammatory responses at implantation sites^28–31^.

Under normal physiological condition, both the fetus and placenta secrete various specialized immune regulatory factors such as IL-10 (Th-2), TGF-β, progesterone, and placental growth hormone to maintain immune tolerance and prevent the cytotoxic effect of NK cell on trophoblastic cells^32,33^. Reduced TGF-β levels have been associated with recurrent miscarriage^34,35^, and successful pregnancy was typically associated with a Th2-dominant immune response, whereas excessive Th1 activity correlated with pregnancy failure^33^. Elevated Th1 cytokines was mentioned to enhance inflammatory and thrombotic responses, increasing the risk of abortion in animal studies^36^.

TGF-β was known as a key cytokine involved in embryonic implantation, trophoblast invasion, placental development, angiogenesis, and fetal growth. TGF-β1 influences the invasion of the uterine wall and spiral arteries that ensure a steady blood supply to the placenta^37^. Consistent with these functions, the reduced TGF-β gene expression observed in the present study was associated with histopathological findings showing poorly developed placental blood vessels.

TGF-β was demonstrated to promote the proliferation of endometrial cells in neoplastic growths, which share a similar mechanism with the invasiveness of trophoblast implantation in normal endometrium cells^38^. Interestingly, phycocyanin pigment of *Spirulina platensis*, was mentioned to have anti-neoplastic activity through inhibition of the TGF-β/SMAD signaling pathway, suggesting its potential as a therapeutic agent in metastatic endometrial cancer^39^.

## Conclusion

This study concluded that administration of nano-spirulina may serve as a promising nutritional strategy as rich source of protein, enhances feed efficiency ratio and elevates the antioxidant markers against heat stress. However, nano-spirulina administration during early pregnancy requires caution, as it was associated with immunomodulation by skewing immunity to type 1 (Th1) which was associated with pregnancy failure, early embryonic loss, fetal resorption, and impaired placental development. These findings highlight a potential reproductive risk. Therefore, further investigations are essential on large scale and on different types of animals to define the risk of its immunmodulation on pregnant animals and to determine optimal doses and timing of supplementation.

The encountered obstacles in the current work were to find the embryo tissue and examine its histopathological picture. Despite demonstrating the enlarged uterus and the placenta as fingerprint of the embryo, we hope to conduct further studies in this respect to confirm these results and obtain the embryo tissue. Also, we advise doing the next work in normal climatic condition without heat stress to study the case in depth. Also, it will be better to use ultrasonography to follow up the fetus’s condition.

## Data Availability

All data of this study are included in this article. The data will be available on request.
